# Variability in surveillance practice for patients with diagnosis of bicuspid aortic valve syndrome

**DOI:** 10.1038/s41598-022-25571-x

**Published:** 2022-12-20

**Authors:** Arianna M. Kahler-Quesada, Ishani Vallabhajosyula, Sameh Yousef, Makoto Mori, Andrea Amabile, Roland Assi, Arnar Geirsson, Prashanth Vallabhajosyula

**Affiliations:** 1 Division of Cardiac Surgery, Yale Aortic Institute, Yale University School of Medicine, 330 Cedar Street, Boardman 204L, New Haven, CT 0652 USA; 2 Center for Outcomes Research and Evaluation, Yale-New Haven Hospital, New Haven, CT USA

**Keywords:** Outcomes research, Cardiology

## Abstract

In patients with bicuspid aortic valves, guidelines call for regular follow-up to monitor disease progression and guide intervention. We aimed to evaluate how closely these recommendations are followed at a tertiary care center. Among 48,504 patients who received echocardiograms (2013–2018) at a tertiary care center, 245 patients were identified to have bicuspid aortic valve. Bivariate analyses compared characteristics between patients who did and did not receive follow-up by a cardiovascular specialist. During a median follow-up of 3.5 ± 2.2 years (mean age 55.2 ± 15.6 years, 30.2% female), 72.7% of patients had at least one visit with a cardiovascular specialist after diagnosis of bicuspid aortic valve. These patients had a higher proportion of surveillance by echocardiogram (78.7% vs. 34.3%, p < .0001), CT or MRI (41.0% vs. 3.0%, p < .0001), and were more likely to undergo surgery. Patients with moderate-severe valvular or aortic pathology were not more likely to be followed by a specialist or receive follow-up echocardiograms. Follow-up care for patients with bicuspid aortic valve was highly variable, and surveillance imaging was sparse despite guidelines. There is an urgent need for mechanisms to monitor this population with increased risk of progressive valvulopathy and aortopathy.

## Introduction

Bicuspid aortic valve (BAV) is the most common congenital heart disease, with a prevalence of 0.5–1-2% and a slight male predominance^1–5^. Many patients with BAV are asymptomatic and often present in adulthood as an incidental finding on echocardiogram. While survival in adult patients with BAV may not differ significantly from that of the general population (potentially due to the efficacy of AVR and similar interventions)^3,6–8^, patients with BAV are at an increased risk for various aortic pathologies including aortic stenosis (AS), aortic regurgitation (AR), aortic root dilation, aortic aneurysm, and aortic dissection^1,3,9^. A systematic review of 11,000 patients reported that aortic aneurisms were present in 20–40% of patients with BAV, though less than 0.5% suffered a dissection^5^. Other studies report that up to 84% of patients with BAV may eventually develop an aortic aneurysm over the course of their lifetime, though less than 5% will have an aortic dissection^10,11^. The risk of these pathologies has prompted guidelines for surveillance of patients with BAV in order to guide timely intervention.

The 2018 American Association for Thoracic Surgery (AATS) guidelines for the management of BAV recommend serial evaluations of the aorta by transthoracic echocardiogram (TTE) with intervals tailored to the presence and severity of aortic dilation^9^. The 2020 American College of Cardiology/American Heart Association (ACC/AHA) guidelines suggest lifelong surveillance in patients with aortic dilation ≥ 4.0 cm, and MRI or CT evaluation of difficult to assess structures^12^. Additionally, the decision to pursue surgery is often based on the severity of valvular pathologies^7^, for which the American Society of Echocardiography gives specific guidelines^13^. Overall, all major cardiology and cardiac surgery societies recommend careful surveillance in BAV patients.

Although there is consensus on the need to carefully follow these patients, it is unknown how well current guidelines are adopted into clinical practice for incidentally detected BAV. Therefore, we aimed to understand the extent of the clinical gap in the implementation of guideline-based surveillance for BAV patients at a tertiary care health system.

## Materials and methods

### Patient population

This was a single center retrospective study of adult patients with bicuspid aortic valve diagnosed by inpatient or outpatient transthoracic echocardiogram (TTE) or transesophageal echocardiogram (TEE) between 2013 and 2018 at a tertiary care center. Yale Institutional Review Board approved this study and individual consent was waived (IRB ID: 2000020356). All methods were performed in accordance with the relevant guidelines and regulations.

**Figure 1:**
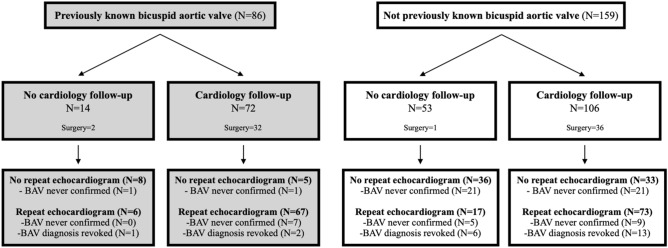
Follow-up patterns in patients diagnosed with bicuspid aortic valve by echocardiogram. Flow-chart of patient cardiology follow-up and echocardiogram studies following initial ECHO (2013–2018). Follow-up was determined if there was at least one recorded appointment with outpatient cardiology or outpatient cardiothoracic surgery following the initial ECHO in this study. Recorded follow-up lasted until January 2020. Unconfirmed BAV diagnosis was determined if the ECHO described the aortic valve as “cannot rule out bicuspid valve”, “possible bicuspid valve”, or “unclear if bicuspid” and further ECHO studies did not clarify. BAV reversal was determined if a follow-up echocardiogram stated “tricuspid aortic valve”.

Among 48,504 unique patients who underwent echocardiogram during that time period, 245 adult patients were identified to have BAV. Patients were identified by screening for the words “bicuspid aortic valve”, “the valve has two cusps”, or “bicuspid valve” in the echocardiogram report. The report was manually reviewed for each patient to confirm the case definition. We recorded cases where the report was equivocal for the diagnosis of bicuspid valve and used the term “possible bicuspid aortic valve” (Fig. 1). If the diagnosis was made prior to 2013, it was classified as “previously known”. The final date of follow-up for chart review was January 23, 2020.

### Collected data and outcomes

The following patient data were collected: demographics, comorbidity, cardiology follow-up, follow-up imaging studies, aortic diameter, the presence of other valvular pathologies, and whether the patient underwent aortic or aortic valve surgery during the study time period. Imaging studies included echocardiogram, CT and MRI. CT and MRI studies were included if the indication was to evaluate the aortic valve or aorta. Dilated aorta was defined as > 40 mm at the aortic root or ascending aorta. Cardiovascular specialist follow-up was defined as at least one outpatient cardiology or cardiothoracic surgery visit following the initial echocardiogram. Data collected only reflects what was captured in our health system.

To characterize follow-up patterns, we compared patients who received cardiovascular specialist follow-up to those who did not. In order to characterize how patients were followed based on aortic and valvular pathology, we also compared follow-up patterns between patients with normal versus dilated aortic diameters at initial presentation, as well as between patients with varying levels of aortic stenosis or regurgitation.

### Statistical analysis

Chi-squared analysis for categorical variables and two-tailed t-tests for continuous variables were used to evaluate whether patient and echocardiographic characteristics differed between patients who were diagnosed with bicuspid aortic valve by echocardiogram. P value of < 0.05 defined statistical significance. We used GraphPad Prism for analysis (version 8, GraphPad Software, San Diego, CA).

## Results

The mean age of the cohort was 55.2 ± 15.6 years and 30.2% were female. In this study, the prevalence of incidental BAV was much lower than what is reported in the general population (0.05% vs 0.5–2%)^1–5^. During a median follow-up of 3.5 ± 2.2 years, 72.7% of patients had at least one visit in an outpatient cardiovascular clinic after the initial diagnosis of bicuspid aortic valve by echocardiogram. Patients followed by a cardiovascular specialist had a higher likelihood of receiving at least one follow-up echocardiogram (78.7% vs. 34.3%, p < 0.0001), at least one CT or MRI (41.0% vs. 3.0%, p < 0.0001), and were more likely to undergo corrective surgery (39.3% vs 4.5%, p < 0.0001), compared with patients not followed by a cardiovascular specialist. Among patients who were followed by a cardiovascular specialist, the average duration between echocardiograms was 1.11 ± 0.98 years. In addition, the mortality of patients was lower when followed by a cardiovascular specialist (6.7% vs 23.9%, p < 0.001) (Table 1).

**Table 1:** Demographics of patients who received cardiology follow-up.

Patient characteristics	All patients (N = 245)				P value
	Not followed by cardiac specialist (n = 67)		Followed by cardiac specialist (n = 178)		
	*N*	*%,* [*SD*]	*N*	*%,* [*SD*]	
Age at presentation, mean	56	[18.4]	54.8	[14.4]	0.21
Male	48	[19.6]	123	[50.2]	0.76
**Race**					
White	51	76.1%	142	79.8%	0.80
African American	9	13.4%	19	10.7%	
Other	7	10.4%	17	9.6%	
Follow-up at institution (yrs)	2.24	[2.3]	3.98	[2.1]	** < 0.0001**
**Studies**					
BAV previously known	14	20.9%	72	40.4%	**0.006**
Follow-up echocardiogram	23	34.3%	140	78.7%	** < 0.001**
Follow-up CT or cMRI	2	3.0%	73	41.0%	** < 0.001**
**Comorbidities**					
Hypertension	40	59.7%	98	55.1%	0.41
Coronary artery disease	10	14.9%	36	20.2%	0.36
BMI, mean (SD)	28.2	[7.3]	29.3	[6.7]	0.2
Family history of heart disease	21	31.3%	79	44.4%	0.038
**Conditions at initial presentation**					
Aortic stenosis or regurgitation (mod-severe)	24	35.8%	80	44.9%	0.24
Ascending aorta (diameter, cm) mean	3.71	[0.7]	3.94	[0.7]	0.039
Aortic root (diameter, cm) mean	3.41	[0.7]	3.45	[0.6]	0.42
Aortic root ≥ 3.5 cm	23	34.3%	78	43.8%	0.22
Ejection Fraction at initial echo, mean	58.2	[10.6]	57.7	[11.5]	0.74
Underwent surgery (valve, root, and/or ascending aorta)	3	4.5%	68	39.3%	** < 0.001**
Overall mortality	16	23.9%	12	6.7%	** < 0.001**

*BAV*=bicuspid aortic valve, *BMI*=body mass index, *CT*=computerized tomography, *echo*=echocardiogram, *cMRI*=cardiac magnetic resonance imaging, *SD*=standard deviation, *yrs*=years.

Significant values are in bold.

Thirty-five percent (N = 86) of patients in our study had a previously known BAV (as per their medical records), while the rest were given their diagnoses during the study period. In patients with a previous diagnosis of BAV, 84% (N = 72) were followed by a cardiovascular specialist, while 67% (N = 106) of new diagnoses were followed. In addition, many of the patients who had a potential but unclear BAV diagnosis never received a follow-up ECHO in order to confirm or deny the diagnosis (17.9%, N = 44), or never received a firm diagnosis even after multiple echocardiograms (8.6%, N = 21) (Fig. 1). Furthermore, follow-up echocardiograms did not always provide both aortic dimensions (aortic root or ascending aorta diameter) and mean valvular gradients (N = 109, 67% of the final echocardiograms in the study period) and only 16% (N = 40) of patients ever received a report on the orientation of their bicuspid valve (Type 0 = 2, Type 1 = 36, Type 2 = 1).

Patients were then stratified by aortic diameter and valve. Patients with dilated aorta were not more likely to receive cardiovascular specialist follow-up. However, they were more likely to receive a follow-up echocardiogram (74.2% vs. 61.8%, p = 0.047), a CT or MRI (44.1% vs. 22.4%, p = 0.0003), and surgery (41.9% vs. 21.1%, p = 0.0005) than patients with normal aortic diameters (Table 2).

**Table 2:** Follow-up patterns of patients based on initial valve or aortic dysfunction.

Patient characteristics	All patients (N = 245)				P value
	None to mild aortic stenosis or regurgitation		Moderate or severe aortic stenosis or regurgitation		
	*N*	*%,* [*SD*]	*N*	*%,* [*SD*]	
	141	57.6%	104	42.4%	
Age at presentation, mean (SD)	55.2	[15.7]	55.2	[15.4]	0.97
Male	98	69.5%	73	70.2%	0.91
**Follow-up**					
Followed by cardiac specialist	98	69.5%	80	76.9%	0.20
Follow-up echocardiogram	89	63.1%	74	71.2%	0.19
Follow-up CT or Cmri	36	25.5%	39	37.5%	**0.04**
Underwent surgery	17	12.1%	54	51.9%	** < 0.001**

	Normal aortic diameters at initial echo		Patients with initial aortic dilatation (> 40 mm)		
	*N*	*%,* [*SD*]	*N*	*%,* [*SD*]	
	152	62.0%	93	38.0%	
**Follow-up**					
Followed by cardiac specialist	107	70.4%	71	76.3%	0.31
Follow-up echocardiogram	94	61.8%	69	74.2%	**0.047**
Follow-up CT or cMRI to evaluate	34	22.4%	41	44.1%	** < 0.001**
Underwent surgery	32	21.1%	39	41.9%	** < 0.001**

*BAV*=bicuspid aortic valve, *CT*=computerized tomography, *echo*=echocardiogram, c*MRI*=cardiac magnetic resonance imaging, *Stdev or SD*=standard deviation, *yrs*=years.

Significant values are in bold.

In a similar vein, patients with moderate to severe aortic valve dysfunction (stenosis and/or regurgitation) were not more likely to have more frequent follow-up than patients with none-mild aortic valve dysfunction. Patients with moderate to severe aortic valve dysfunction were not more likely to be followed by a specialist (76.9% vs. 69.5%, p = 0.198) or receive a follow-up echocardiogram (71.2% vs. 63.1%, p = 0.188). However, they were more likely to receive a CT or MRI to evaluate the aorta or aortic valve (37.5% vs. 25.5%, p = 0.045) and/or undergo surgery (51.9% vs. 12.1%, p < 0.0001) (Table 2).

We assessed the impact of clinical follow-up by cardiovascular specialist on timely surgical intervention. Overall, 28.9% (N = 71) of patients underwent aortic and/or aortic valve surgery, 47.9% (N = 34) of whom had a previously known diagnosis of BAV. Among surgical patients, 95.8% (N = 68) were followed by a cardiovascular specialist, 88.7% (N = 63) received at least one follow-up echocardiogram, and 60.6% (N = 43) received a CT or MRI to evaluate the aorta or aortic valve. The most common indications for surgery were ascending aortic aneurysm with or without stenosis or regurgitation (N = 25, 34%), aortic stenosis (N = 24, 34%), aortic regurgitation (N = 10, 14%), and endocarditis with or without stenosis or regurgitation (N = 5, 7%). After surgery for BAV syndrome, 85.9% (N = 61) received at least one repeat echocardiogram, and 35.2% (N = 25) received at least one CT or MRI for the purpose of surveillance (Table 3).

**Table 3:** Demographics of patients who underwent surgery.

Variables	All patients (N = 245)	
	Received surgery	
	N	*%,* [*SD*]
	71	29.0%
Age at time of surgery, mean (SD)	54.5	[13.1]
Male	51	71.8%
**Race**		
White	61	85.9%
African American	3	4.2%
Other	7	9.9%
Previously known BAV	34	47.9%
**Post-surgery studies**		
Follow-up echocardiogram	61	85.9%
Follow-up CT or cMRI	25	35.2%
**Type of surgery**		
AVR	38	53.5%
Valve and ascending aorta	12	16.9%
Valve and root	6	8.5%
Valve and root and ascending aorta	8	11.3%
Ascending aorta repair	5	7.0%
TAVR	2	2.8%
**Indication for surgery**		
Aortic stenosis	24	33.8%
Aortic regurgitation	10	14.1%
AS and AR	1	1.4%
Ascending aortic aneurysm	16	22.5%
Ascending aortic aneurysm with AS and/or AR	9	12.7%
Endocarditis	2	2.8%
Endocarditis with AR and/or AS	3	4.2%
Thoracic aneurysm	1	1.4%
Aortic disease	2	2.8%
No data	3	4.2%
Need for a second surgery	1	1.4%
Overall mortality	2	2.8%

*AS*=aortic stenosis, *AR*=aortic regurgitation, *AVR*=aortic valve replacement, *BAV*=bicuspid aortic valve, *CT*=computerized tomography, *echo*=echocardiogram, *cMRI=*cardiac magnetic resonance imaging, *Stdev or SD*=standard deviation, *TAVR*=transcatheter aortic valve replacement.

## Discussion

In this study, follow-up care for patients with a diagnosis of bicuspid aortic valve was highly variable. Current guidelines from the The American Association for Thoracic Surgery state that the interval for follow-up imaging should be based on severity of disease (especially aortic dilation)^9^, and research has shown that bicuspid aortic valves will often progress in severity as patients age^1,3,8^. Therefore, once patients are diagnosed with BAV (or have a possible BAV found by echocardiogram), guidelines recommend that they should be followed by a cardiovascular specialist in order to determine the best schedule for imaging and/or surgical intervention.

In this study, we found that over the mean follow-up of 3.5 years, more than a quarter of patients with a bicuspid aortic valve were never seen by a cardiovascular specialist after diagnosis. In addition, a third of patients did not receive a follow-up echocardiogram, and less than half of the patients who may have benefitted from CT or MRI surveillance according to some guidelines (2018 AATS) received it**.** Furthermore, many unclear bicuspid diagnoses, such as those labeled as “possible BAV” or “unable to rule out BAV”, did not receive a follow-up echocardiogram and/or a firm diagnosis following the initial echocardiogram. These data beg the question of how the pathology of those patients progressed, and whether they would have benefitted from earlier intervention or acknowledgement of the potential complications of BAV. It is interesting to note that while mortality was found to be significantly less in patients who were followed clinically, this study cannot draw any direct correlations. However, this data may be an indication of the type and frequency of the care those patients received care more generally.

Current guidelines suggest that the frequency and type of surveillance should be based on severity of aortic dilatation. Specifically, the 2018 AATS guidelines push for comprehensive serial evaluation. After the initial evaluation of the valve morphology, these guidelines state that normal aortic diameters should receive echocardiogram surveillance every 3–5 years, stable aortic dilation (40-49 mm) should be evaluated every 2–3 years (after an initial check at 12 months), and more advanced aortic dilation (> 50 mm) should be imaged yearly. It is also further recommended that aortic dilation > 40 mm should be investigated by echocardiogram-gated MRI or CT angiography^9^. ACC/AHA guidelines suggest a slightly more flexible pattern of surveillance^14,15^, with 2020 ACC/AHA guidelines suggesting MRI/CT for difficult to assess structures, then lifelong surveillance of patients whose aortic diameter ≥ 4.0 cm, with intervals determined by family history and progression rate. These guidelines also suggest considering a screening TTE in the first-degree relatives of patients with BAV^12^. These guidelines state that TTE is usually adequate for hemodynamics and evaluation of anatomy, while TEE can provide improved 2D and 3D images. Cardiac MRI or CT provides better images of the aorta (including the sinotubular junction, sinuses, or ascending aorta) when both of those imaging modalities are not adequate to evaluate valve and aortic morphology.

In our study, while patients with aortic dilation of > 40 mm were more likely to receive follow-up imaging, they were not more likely to have outpatient specialist follow-up. Furthermore, the severity of valvular disease at presentation (aortic stenosis or regurgitation) did not significantly affect clinical follow-up or imaging surveillance patterns. (Table 2).

Patients with BAV are at risk for aortic dilation independent of valvular dysfunction, even beginning in childhood^16^, and aortic dilation can progress even with normally functioning valves^17,18^. At the same time, valvular dysfunction (especially aortic stenosis) is an independent risk factor for dissection^6^. It would follow that severity of disease should impact the level of surveillance by cardiovascular specialists so that both patients and providers can be aware of risks and potential complications over time and manage imaging appropriately. Unfortunately, we found that increased severity of disease did not seem to lead to increased follow-up by a specialist.

Our study speaks to the stark gap in adoption of guidelines and ensuring optimal implementation in the clinical setting. They also provide a window of opportunity to improve system wide screening and institution of diagnosis triggered alerts to the right clinical practices so BAV patients are provided optimal care. This gap in quality of care attests to the importance of interdisciplinary communication between cardiology, radiology, and cardiac surgery.

This study has the following limitations. Due to the retrospective nature of this study, the availability of information such as previously known diagnoses were limited by explicit documentation in available notes. In addition, this was a single-center study, which limits generalizability and raises the possibility of not capturing outside imaging or follow-up in our analysis, although extensive search was conducted using our electronic medical record system. Of note, this study found a much lower prevalence of bicuspid aortic valve than what has been reported in the general population. Various phrases were used to identify echocardiogram reports with a mention of bicuspid aortic valve, but it is possible that not all instances of BAV were captured due to variations in the wording of reports.

Overall, follow-up and use of surveillance imaging of the aorta or the aortic valve may be variable despite awareness of guideline recommendations. There is an urgent need for systematic surveillance and implementation of clinical follow-up mechanisms to monitor this patient population with increased risk of progressive valvulopathy and aortopathy.

## Data Availability

The datasets generated during and/or analyzed during the current study are available from the corresponding author on reasonable request.

## References

[1] Hoffman JI, Kaplan S (2002). The incidence of congenital heart disease. J. Am. Coll. Cardiol..

[2] Movahed M-R, Hepner AD, Ahmadi-Kashani M (2006). Echocardiographic prevalence of bicuspid aortic valve in the population. Heart Lung Circ..

[3] Siu SC, Silversides CK (2010). Bicuspid aortic valve disease. J. Am. Coll. Cardiol..

[4] Larson EW, Edwards WD (1984). Risk factors for aortic dissection: A necropsy study of 161 cases. Am. J. Cardiol..

[5] Masri A, Svensson LG, Griffin BP, Desai MY (2017). Contemporary natural history of bicuspid aortic valve disease: A systematic review. Heart.

[6] Michelena HI, Desjardins VA, Avierinos JF, Russo A, Nkomo VT, Sundt TM (2008). Natural history of asymptomatic patients with normally functioning or minimally dysfunctional bicuspid aortic valve in the community. Circulation.

[7] Michelena HI, Khanna AD, Mahoney D, Margaryan E, Topilsky Y, Suri RM (2011). Incidence of aortic complications in patients with bicuspid aortic valves. JAMA.

[8] Tzemos N, Therrien J, Yip J, Thanassoulis G, Tremblay S, Jamorski MT (2008). Outcomes in adults with bicuspid aortic valves. JAMA.

[9] Borger MA, Fedak PWM, Stephens EH, Gleason TG, Girdauskas E, Ikonomidis JS (2018). The American Association for Thoracic Surgery consensus guidelines on bicuspid aortic valve-related aortopathy: Full online-only version. J. Thorac. Cardiovasc. Surg..

[10] Michelena HI, Prakash SK, Della Corte A, Bissell MM, Anavekar N, Mathieu P (2014). Bicuspid aortic valve: Identifying knowledge gaps and rising to the challenge from the International Bicuspid Aortic Valve Consortium (BAVCon). Circulation.

[11] Ward C (2000). Clinical significance of the bicuspid aortic valve. Heart.

[12] Writing Committee Members, Otto, C.M., Nishimura, R.A., Bonow, R.O., Carabello, B.A., Erwin, J.P. 3rd, Gentile, F. *et al*. 2020 ACC/AHA guideline for the management of patients with valvular heart disease: A report of the American College of Cardiology/American Heart Association Joint Committee on Clinical Practice Guidelines. *J. Am. Coll. Cardiol*. **77**(4), e25–e197 (2021). 10.1016/j.jacc.2020.11.018 33342586

[13] Baumgartner H, Hung J, Bermejo J, Chambers JB, Edvardsen T, Goldstein S (2017). Recommendations on the echocardiographic assessment of aortic valve stenosis: A focused update from the European Association of Cardiovascular Imaging and the American Society of Echocardiography. Eur. Heart J. Cardiovasc. Imaging..

[14] Nishimura RA, Otto CM, Bonow RO, Carabello BA, Erwin JP, Guyton RA (2014). American College of Cardiology/American Heart Association Task Force on Practice Guidelines. 2014 AHA/ACC guideline for the management of patients with valvular heart disease: A report of the American College of Cardiology/American Heart Association Task Force on Practice Guidelines. J. Am. Coll. Cardiol..

[15] Stout KK, Daniels CJ, Aboulhosn JA, Bozkurt B, Broberg CS, Colman JM (2019). 2018 AHA/ACC Guideline for the Management of Adults with Congenital Heart Disease: A Report of the American College of Cardiology/American Heart Association Task Force on Clinical Practice Guidelines. J. Am. Coll. Cardiol..

[16] Beroukhim RS, Kruzick TL, Taylor AL, Gao D, Yetman AT (2006). Progression of aortic dilation in children with a functionally normal bicuspid aortic valve. Am. J. Cardiol..

[17] Davies RR, Kaple RK, Mandapati D, Gallo A, Botta DM, Elefteriades JA (2007). Natural history of ascending aortic aneurysms in the setting of an unreplaced bicuspid aortic valve. Ann. Thorac. Surg..

[18] Kang JW, Song HG, Yang DH, Baek S, Kim DH, Song JM (2013). Association between bicuspid aortic valve phenotype and patterns of valvular dysfunction and bicuspid aortopathy: Comprehensive evaluation using MDCT and echocardiography. JACC Cardiovasc. Imaging.

